# Specifically Progressive Deficits of Brain Functional Marker in Amnestic Type Mild Cognitive Impairment

**DOI:** 10.1371/journal.pone.0024271

**Published:** 2011-09-15

**Authors:** Feng Bai, David R. Watson, Yongmei Shi, Yi Wang, Chunxian Yue, YuhuanTeng, Di Wu, Yonggui Yuan, Zhijun Zhang

**Affiliations:** 1 Medical School of Southeast University, Nanjing, China; 2 The Department of Neurology, Affiliated ZhongDa Hospital and Institute of Neuropsychiatry of Southeast University, Nanjing, China; 3 Computational Neuroscience, University of Ulster, Derry, Northern Ireland, United Kingdom; Federal University of Rio de Janeiro, Brazil

## Abstract

**Background:**

Deficits of the default mode network (DMN) have been demonstrated in subjects with amnestic type mild cognitive impairment (aMCI) who have a high risk of developing Alzheimer’s disease (AD). However, no longitudinal study of this network has been reported in aMCI. Identifying links between development of DMN and aMCI progression would be of considerable value in understanding brain changes underpinning aMCI and determining risk of conversion to AD.

**Methodology/Principal Findings:**

Resting-state fMRI was acquired in aMCI subjects (n = 26) and controls (n = 18) at baseline and after approximately 20 months follow up. Independent component analysis was used to isolate the DMN in each participant. Differences in DMN between aMCI and controls were examined at baseline, and subsequent changes between baseline and follow-up were also assessed in the groups. Posterior cingulate cortex/precuneus (PCC/PCu) hyper-functional connectivity was observed at baseline in aMCI subjects, while a substantial decrement of these connections was evident at follow-up in aMCI subjects, compared to matched controls. Specifically, PCC/PCu dysfunction was positively related to the impairments of episodic memory from baseline to follow up in aMCI group.

**Conclusions/Significance:**

The patterns of longitudinal deficits of DMN may assist investigators to identify and monitor the development of aMCI.

## Introduction

Mild cognitive impairment (MCI) is associated with a high risk for dementia [1], [2]. Amnestic type MCI (aMCI) is generally regarded as a pathologic precursor to Alzheimer's disease (AD), however the considerable clinical and biological heterogeneity in aMCI indicates the possibility of 'reverse conversion' or 'long-term steady state'. Its predominant symptom is episodic memory loss, including aMCI-single domain (the impairment involves only the memory domain) and aMCI-multiple domain (the impairments in the memory domain plus at least one other cognitive domain). In addition, non-aMCI deficits are often conceptualized as the prodromal phase for other causes of dementia [2], but they may also be involved in AD-dementias [3]. In fact, neuropathologic lesions similar to AD have been demonstrated in aMCI.[4]. As 10–15% of aMCI subjects progress to AD annually, the detection of progressive deficits in aMCI that may provide a risk indicator of the liability to convert to AD.

The default mode network (DMN) has been widely explored by task-based deactivation [5]-[7] and resting-state neuroimaging [8]-[10] studies. Multiple behavioral correlates of the DMN also have been evidenced [11], including episodic memory processing, self-referential processing, stream of consciousness, day dreaming, mind wandering, unconstrained thoughts, free association, and monitoring the internal and external environment [12]. Indeed, amyloid deposition at the earliest stages of AD shows a distribution that is remarkably similar to the anatomy of the DMN [13], and the extent of amyloid deposition related to DMN in AD patients was significantly higher than that observed in older controls [12]. Furthermore, increased amyloid deposition has also been associated with aberrant DMN activity [14] and functional connectivity [15] in nondemented older adults. A recent study further revealed that the mechanism underlying the regional vulnerability to amyloid-β(Aβ) deposition in AD relates to the endogenous neuronal activity of DMN regulating the regional concentration of interstitial fluid Aβ, which drives local Aβaggregation [16]. Therefore, AD pathology may be preferentially located throughout the DMN [17]. Moreover, cross-sectional neuroimaging findings have suggested that patients with AD [18]–[26] and aMCI [27]–[31] could be characterized by abnormalities in the DMN system. However, little is known about the progressive deficits of DMN in aMCI subjects.

The objective of the present study was to examine changes in DMN function in aMCI subjects over time. Resting-state fMRI was adopted in the present study as the magnitude of DMN activation has been shown to vary both as a function of task demand and of the participant’s ability to disengage from a task [32]. Independent component analysis (ICA), which can separate independent spatio-temporal patterns of coherent neuronal activity without prior knowledge about activity waveforms or locations [20], was used to investigate to compare the changes of DMN in aMCI compared to matched controls. This approach may provide evidence of the patterns of progressive deficits of DMN in the development of aMCI subjects.

## Materials and Methods

### Participants

The study was approved by the Research Ethics Committee of Affiliated ZhongDa Hospital, Southeast University and written informed consent was obtained from all participants. Firstly, 115 aMCI subjects and 126 healthy controls were recruited from 1480 aging subjects in Chinese community through strict diagnostic criteria. Secondly, 48 aMCI and 36 well-matched healthy recruited subjects underwent the baseline fMRI scan between Aug. 2006 and Feb. 2008. Thirdly, after a mean follow-up period of 20 months (ranging from 15 months to 30 months) between Feb. and Jun. 2009, subjects not completing the testing procedures or otherwise dropping out of the study (i.e. non-responders/refusals/death) were excluded (aMCI n = 14; controls n = 15). Six of the remaining aMCI subjects subsequently developed AD identified by clinical criteria, while no healthy control subject was found to convert to AD. Five subjects (aMCI n = 2; controls n = 3) were further excluded after the evaluation of head motion (i.e. exceeding 3 mm in transition or 30 in rotation) or poor quality of image (i.e. ghost intensity). Therefore, 26 aMCI subjects and 18 matched healthy controls underwent the baseline fMRI scan, with these participants completing a follow-up scan at approximately 20 months, and these groups were matched for follow up length.

### Entry criteria

All subjects underwent diagnostic evaluations including a clinical interview and focused neurological and mental status exam, review of medical history, and demographic inventory. Cognitive functioning was evaluated by a mini mental state examination (MMSE) and the degree of dementia determined by a clinical dementia rating scale (CDR). In addition, a neuropsychological battery that consisted of Auditory Verbal Learning Test (AVLT)-delayed recall, Rey-Osterrieth Complex Figure Test, Digit Span Test, Symbol Digit Modalities Test, Trail Making Test-A and –B, and Clock Drawing Test to evaluate the function of episodic memory, attention, psychomotor speed, executive function and visuo-spatial skills respectively.

### Inclusion criteria

Presence of aMCI (including aMCI-single domain and aMCI-multiple domain) was determined following the procedure of Petersen et al. (1999) [1] and others [33], including (1) subjective memory impairment corroborated by subject and an informant; (2) objective memory performances documented by an AVLT-delayed recall score less than or equal to 1.5 SD of age- and education-adjusted norms (cutoff of ≤ 4 correct responses on 12 items for ≥ 8 years of education); (3) MMSE score of 24 or higher; (4) CDR of 0.5; (5) no or minimal impairment in activities of daily living; (6) absence of dementia, or not sufficient to meet the NINCDS-ADRDA Alzheimer's Criteria (National Institute of Neurological and Communicative Disorders and Stroke and the Alzheimer's Disease and Related Disorders Association). The diagnostic process was conducted by an experienced neuropsychiatrist by structured interview with subjects and their informants.

### Exclusion criteria

Participants were excluded from the study if they had a history of known stroke, alcoholism, head injury, Parkinson’s disease, epilepsy, major depression or other neurological or psychiatric illness, major medical illness, severe visual or hearing loss. Controls were required to have a CDR of 0, MMSE score ≥ 26, and an AVLT-delayed recall score > 4 for those with 8 or more years of education.

### Longitudinal follow-up

Follow-up neuropsychological tests and fMRI parameters were identical to those undertaken at baseline in every participant. Mean follow-up period was twenty months. Diagnostic and Statistical Manual of Mental Disorders-IV (DSM-IV) and NINCDS-ADRDA Alzheimer's Criteria were subsequently used to clinical diagnosis of AD.

### Magnetic resonance imaging procedures

The subjects were scanned using a General Electric 1.5 Tesla scanner (General Electric Medical Systems, USA) with a homogeneous birdcage head coil. Subjects lay supine with the head snugly fixed by a belt and foam pads to minimize head motion. Conventional axial Fast Relaxation Fast Spin Echo sequence (FRFSE) T2 weighted anatomic MR images were obtained to rule out cerebral infarction or other lesions: repetition time (TR) = 3500 ms; echo time (TE) = 103 ms; flip angle (FA) = 90^0^; acquisition matrix  =  320×192; field of view (FOV)  =  240 mm×240 mm; thickness  =  6.0 mm; gap  =  0 mm; no. of excitations  =  2.0. High-resolution T1-weighted axial images covering the whole brain were acquired using a 3D spoiled gradient echo (SPGR) sequence as follow: TR = 9.9 ms; TE = 2.1 ms; FA = 15^0^; acquisition matrix  =  256×192; FOV  =  240 mm×240 mm; thickness  =  2.0 mm; gap  =  0 mm. The functional scans (T2* weighted images) involved the acquisition of 30 contiguous axial slices using a gradient-recalled echo-planar imaging (GRE-EPI) pulse sequence: TR = 3000 ms; TE = 40 ms; FA = 90^0^; acquisition matrix  =  64×64; FOV = 240 mm×240 mm; thickness  =  4.0 mm; gap  =  0 mm and 3.75×3.75 mm^2^ in-plane resolution parallel to the anterior commissure–posterior commissure line. This acquisition sequence generated 142 volumes in 7 min and 6 s. All subjects have eyes closed during scanning. It should be noted that the same parameters were employed in both baseline and follow-up scans, and two experienced radiologists executed the scans in the whole longitudinal process.

### Image preprocessing

Data analyses of four groups were conducted with SPM5 (www.fil.ion.ucl.ac.uk/spm) using the same procedures. The first eight volumes of the scanning session were discarded to allow for T1 equilibration effects. The remaining images were corrected for timing differences and motion effects. Participants with head motion more than 3 mm maximum displacement in any direction of x, y, and z or 3 degree of any angular motion were excluded. The resulting images (both baseline and follow-up data) were spatially normalized into the SPM5 Montreal Neurological Institute (MNI) echo-planar imaging template using the default settings and resampling to 3×3×3 mm^3^ voxels, and smoothed with a Gaussian kernel of 8×8×8 mm.

### Independent component analysis

ICA was applied to fMRI data of each subject by using GIFT (Version 1.3b; http://icatb.sourceforge.net). This involves a preliminary dimension estimation on each of four groups to determine the number of independent components (ICs), using the minimum description length (MDL) criterion [28], [34]–[36]. The fMRI data of each group were further analyzed separately according to previous studies [28], [35], [37], [38]. This step is associated with two reasons: one is to ensure that the resting-state networks have similar spatial pattern in these four groups; on the other hand, “breakdown” of function connectivity in resting-state networks are associated with in patients. This breakdown is thought to be a reduction in connectivity, while change the topology of resting-state networks and eventually produces new networks. It cannot be assumed that these datasets of patients and controls have the same number of ICs, and it is thus appropriate to apply MDL separately on the present four datasets. In the present study, there were respectively 33/31/35/35 ICs for the data of baseline aMCI group, follow-up aMCI group, baseline controls group, and follow-up controls group. Briefly, three stages were further performed: considering all subjects gather into ICA mode, a reasonable assumption in many fMRI studies due to the large number of time points often acquired. Principle component analysis (PCA) was used to reduce the data within a lower dimensionality; estimation of independent sources was performed using the Infomax algorithm; back reconstruction, consisting of computing individual subject image maps and time courses, followed by component grouping across subjects and thresholding the resulting group ICA images [39] Then, ICs for each subject were obtained. The use of the ICA was assumed to reflect global functional connectivity. In order to display the voxels that contributed most strongly to a particular IC, the intensity values in each spatial map were converted to Z-values, removing the average value and dividing by the standard deviation of the intensity distribution [39], [40]. Each voxel within IC of single subject showed a Z score that represents the degree of correlationship between this voxel’s time series and the mean time series of that particular IC. As ICA on fMRI data intrinsically extracts patterns of coherent neuronal activity (i.e. networks), it is commonly accepted that Z values can provide an indirect measure of functional connectivity [36], [37], [41]. In the current study, the regions only within the brain were considered, i.e., background and other tissues outside the brain were removed.

An implemented in the GIFT software [39], the components to be retained for further analysis among the 33/31/35/35 estimated ICs for four groups were selected based on the largest spatial correlation [42]–[44] with the specific DMN template [36], [37]. This template was provided by Dr. Liao (Key Laboratory for Neuroinformation of Ministry of Education, School of Life Science and Technology, University of Electronic Science and Technology of China), which has been recruited in previous studies (35,36). In particular, this template mainly included posterior cingulate cortex/precuneus (PCC/Pcu), bilateral inferior parietal gyrus, angular gyrus, middle temporal gyrus, superior frontal gyrus and medial frontal gyrus. After combination with the spatial correlation for the DMN selection criterion, the resulting IC based on the specific DMN template was consistent with our prior knowledge on this network.

### Voxelwise-based gray matter volume correction

To control for possible DMN differences that may be explained by differences in gray matter distribution between subjects, we included estimates of a voxel’s likelihood of containing gray matter as a covariate (nuisance variable) in the analysis of the resting-state functional data [45]. The purpose of this method is to isolate the functional changes component which cannot be attributed to anatomical difference and is thus likely due to genuine functional differences. Firstly, Voxel-Based Morphometry (VBM) [46], [47] was used to explore gray matter volume maps of every subject. These maps were transformed into the same standard space as the resting-state fMRI images using affine linear registration [48]. As VBM results can be sensitive to the size of the smoothing kernel used to smooth the tissue segment images, the criterion used in this work was to match the smoothness of the gray matter volume map data to that of the corresponding functional data (8 mm). Finally, the resulting voxelwise gray matter volume maps were input as covariates in the analysis of functional data. The voxelwise-based gray matter volume correction was used for each subject. It noted that one of twenty-six baseline aMCI subjects had no anatomical images. The DMN ICs corrected by voxelwise-based gray matter volume were then analyzed in following process.

### Group-level analyses of the DMN

Within group: to determine the patterns of DMN in each of four groups, the spatial maps of DMN IC in each group were submitted to a random-effect analysis using one-sample t-tests. The thresholds were set at a corrected *P*<0.05, determined by Monte Carlo simulation for multiple comparison (Parameters were: single voxel *P* value  =  0.005, a minimum cluster size of 1242 mm^3^, FWHM  =  8 mm, with mask. See program AlphaSim by D. Ward, and http://afni.nimh.nih.gov/pub/dist/doc/manual/AlphaSim.pdf).

Between groups: (1) a mixed ANOVA with a within-subject repeated factor (time points: baseline and follow up) and an across-subject factor (groups: aMCI and control) with subject as a nested random variable within group was performed. (2) Post hoc test: to explore the details of longitudinal changes in the patterns of DMN IC were in groups, a comparison between aMCI group and controls group at baseline, and a direct comparison between the change estimates between aMCI group (from baseline to follow-up) and controls group (from baseline to follow-up) were further explored. It should be noted that post hoc analyses were masked with a map from groups×time points interaction of aforementioned ANOVA. The thresholds were set at a corrected *P*<0.05, determined by Monte Carlo simulation for multiple comparison (Parameters were: single voxel *P* value  =  0.005, a minimum cluster size of 1242 mm^3^, FWHM  =  8 mm, with mask. See program AlphaSim by D. Ward, and http://afni.nimh.nih.gov/pub/dist/doc/manual/AlphaSim.pdf).

To further explore the role of the changes of DMN IC, firstly, the overlap of aMCI-related baseline changes and longitudinal changes of DMN IC identified via comparison across groups was extracted as region of interest. Secondly, a direct functional index of overlap regions was calculated in all four groups. Finally, we performed a correlative analysis between longitudinal changes in neuropsychological test scores and longitudinal changes of these overlap regions in groups. The thresholds were set at a *P*<0.05.

## Results

### Neuropsychological data

Healthy subjects displayed levels of cognitive performance within the normal range both at baseline and follow up. Compared to controls, aMCI subjects showed deficits in CDR, MMSE, and performance on AVLT-delayed recall and Rey-Osterrieth Complex Figure test-delayed recall (evaluate the function of episodic memory) both at baseline and follow up. Impaired performance on the Trail Making Test and Symbol Digit Modalities Test (evaluate the function of attention, psychomotor speed and executive function) were also observed in the aMCI group at baseline, confirming episodic memory impairment as a predominant symptom in these subjects (i.e. higher effect size than with other cognitive measures). aMCI patients did show stable CDR and MMSE scores suggesting little change in general cognitive abilities over the study period (details see Table 1).

**Table 1:** Demographic and neuropsychological data between aMCI group and healthy controls group.

Items	Baseline				Follow up			
	aMCI group (n = 26)	controls group (n = 18)	*P* (MWU)	Effect size	aMCI group (n = 26)	controls group (n = 17)△	*P*(MWU)	Effect size
Age (years)	71.4±4.3	70.3±4.7	0.357	-	-	-	-	-
Education levels (years)	13.8±2.8	15.1±3.1	0.084	-	-	-	-	-
Gender (male: female)	19∶7	10∶8	0.233	-	19∶7	9∶8	0.233	-
Clinical dementia rating (CDR)	0.5	0	-	-	0.5	0	-	-
Mini mental state exam (MMSE)	27.2±1.5	28.3±1.3	0.026*	0.76	27.2±2.1	28.6±1.8	0.01*	0.69
Auditory verbal memory test- delayed recall	2.8±1.2	8.1±1.9	0.000*	3.42	4.2±2.2	8.6±2.7	0.000*	1.79
Rey-Osterrieth complex figure test	32.9±4.7	34.7±1.4	0.471	0.47	34.0±2.9	35.0±0.8	0.715	0.42
Rey-Osterrieth complex figure test-delayed recall	12.0±7.4	17.3±6.8	0.017*	0.73	12.9±7.6	20.9±7.4	0.002*	1.04
Trail making test-A (seconds)Trail making test-B (seconds)	88.7±36.2182.3±70.4	70.0±28.7139.3±39.2	0.049*0.046*	0.550.71	95.1±44.3175.6±82.8	75.7±26.0138.8±55.1	0.0960.099	0.500.49
Symbol digit modalities test	27.7±10.4	34.3±8.7	0.043*	0.66	29.0±11.4	33.2±13.0	0.164	0.34
Clock drawing test	8.5±1.6	8.9±1.2	0.231	0.27	8.8±2.0	9.1±0.9	0.689	0.17
Digit span test	12.2±2.0	13.2±1.8	0.09	0.51	12.7±2.0	13.6±2.5	0.303	0.40

Values are mean ± (SD); MWU: Mann-Whitney U-test, which was used here due to the neuropsychological data were not normally distributed;

*indicates had statistical difference between groups, *P*<0.05. Δ: One subject of follow-up neuropsychological data in healthy controls was absent. Effect size for distinguishing groups using Hedges g scores, accounting for sample sizes.

### Group ICA

**Figure 1:**
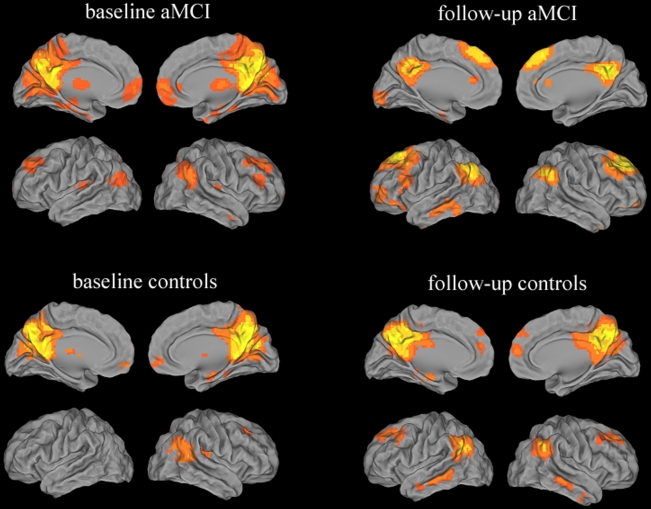
Validation of the ICA approach in aMCI subjects and healthy controls. Images showed the DMN in aMCI group and controls group at baseline and follow up, separately. Significant consistency between these studies is demonstrated across the majority of clusters including the posterior cingulated cortex, precuneus, inferior parietal lobule, prefrontal cortex, ventral anterior cingulate cortex, lateral temporal cortex. Thresholds were set at a corrected *P*<0.05, determined by Monte Carlo simulation.

Within group: the DMN was identified in each of the four groups by Group ICA (Figure 1) and is consistent with previous work in aMCI subjects [28]. A qualitative visual inspection of functional connectivity in DMN, showing the majority of clusters with high consistency in these groups, such that PCC/Pcu, inferior parietal lobule, prefrontal cortex, ventral anterior cingulate cortex and lateral temporal cortex.

**Figure 2:**
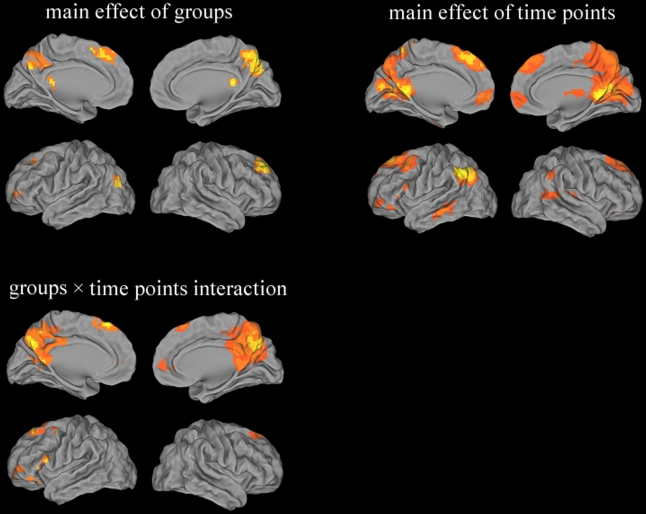
Groups × time points ANOVA of DMN IC functional connectivity. Thresholds were set at a corrected *P*<0.05, determined by Monte Carlo simulation.

Between groups: (1) main effect of groups were in parietal cortex (bilateral PCC/Pcu), frontal cortex (right superior/left middle gyrus) and temporal cortex (left middle gyrus), while main effect of time points were widely observed in parietal cortex (bilateral PCC/Pcu, bilateral inferior parietal lobule), frontal cortex (bilateral medial/ right middle gyrus, bilateral anterior cingulate) and temporal cortex (right superior/ left middle/ bilateral inferior gyrus). In particular, regions associated with groups×time points interaction were parietal cortex (bilateral PCC/Pcu) and frontal cortex (bilateral medial/left middle/ left inferior gyrus, bilateral anterior cingulate) (details see Table 2 and Figure 2).

**Table 2:** Groups × time points ANOVA of DMN IC functional connectivity.

Brain region	BA	Peak MNI Coordiates x, y, z (mm)	PeakF value	cluster size
**(1) main effect of groups**				
B Posterior Cingulate Cortex/ Precuneus	7/30/31	6 -75 48	23.44	19521
R Superior Frontal Gyrus	8/9	21 39 42	19.59	5157
L Middle Frontal Gyrus	6/8/10	-27 0 54	23.62	8154
L Middle Temporal Gyrus	19	-39 -78 18	26.10	1944
**(2) main effect of time points**				
B Posterior Cingulate Cortex/ Precuneus	7/18/30/31	9 -48 6	102.35	87696
B Medial Frontal Gyrus	6/8/9	-6 30 45	51.72	50571
R Middle Frontal Gyrus	11	42 48 -9	42.58	2538
B Anterior Cingulate	10/32	-3 48 0	42.12	6507
R Superior Temporal Gyrus	22	57 -39 9	31.39	7479
L Middle Temporal Gyrus	21	-60 -33 -12	33.36	4779
L Inferior Temporal Gyrus	20	-51 -9 -42	25.41	1998
R Inferior Temporal Gyrus	20	51 -6 -45	23.33	2025
L Inferior Parietal Lobule	39/40	-54 -57 39	78.39	19683
R Inferior Parietal Lobule	39/40	63 -51 42	19.90	3294
**(3) groups×time points interaction**				
B Posterior Cingulate Cortex/ Precuneus	7/23/29/30/31	9 -69 36	64.53	66906
B Anterior Cingulate/ Medial Frontal Gyrus	10/32	12 57 6	19.04	3024
B Superior Frontal Gyrus	6/8	-9 27 57	41.70	14202
L Middle Frontal Gyrus	10	-42 39 3	17.61	3132
L Inferior Frontal Gyrus	47	-54 12 15	27.74	4887

Note: A corrected threshold by Monte Carlo simulation at *P*<0.05. R =  right; L =  left; B =  Bilateral; BA =  Brodmann’s area; Cluster size is in mm^3^; MNI: Montreal Neurological Institute.

**Figure 3:**
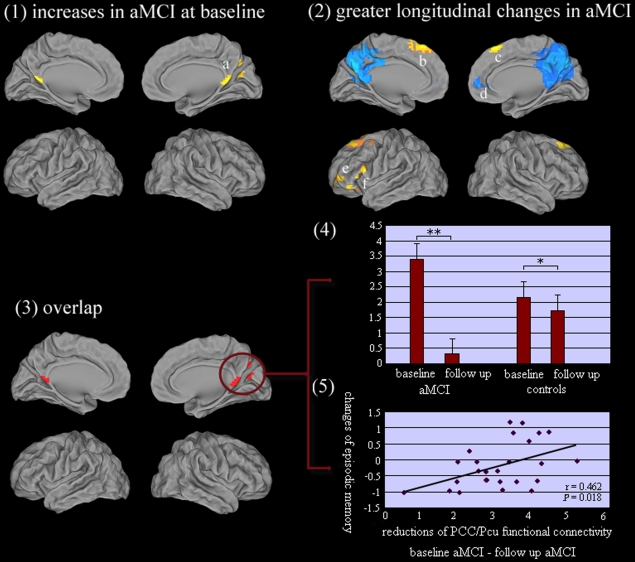
(1). Comparison to controls group at baseline, aMCI group showed increased functional connectivity of DMN IC in a  =  bilateral PCC/PCu. (2). A comparison between the longitudinal changes between aMCI group and controls group, showing a greater decrement in functional connectivity of DMN IC in the aMCI group, particularly in PCC/PCu, and another region was d  =  right anterior cingulate/ medial frontal gyrus. In addition, the longitudinal increased functional connectivity of the prefrontal cortex was observed at follow up in aMCI subjects compared with controls, including b  =  left superior frontal gyrus/ middle frontal gyrus, c  =  right superior frontal gyrus/ middle frontal gyrus, e  =  left middle frontal gyrus and f  =  left inferior frontal gyrus. Thresholds were set at a corrected *P*<0.05, determined by Monte Carlo simulation. (3) Overlap regions (PCC/Pcu) between baseline changes and longitudinal changes of DMN IC were observed in aMCI group compared to controls. (4) Hyper-functional connectivity between the PCC/PCu (overlap regions) and mean DMN IC in baseline aMCI subjects, and significant hypo-connections of these regions were in follow-up aMCI subjects, whilst controls only showed a light decreases at the longitudinal period. * *P*<0.05 (0.046); ** *P*<0.001(0.000). (5) Correlative analysis: within the aMCI group, the decreases of functional connectivity between PCC/PCu and mean DMN IC were positively related to the impairments of episodic memory (AVLT-delayed recall scores, r = 0.462, *P* = 0.018, two-tailed) from baseline to follow up. It should be noted that the raw scores of AVLT-delayed recall for each subject was transformed to z scores.

(2) Post hoc test: As compared to controls group, aMCI group showed increased functional connectivity of DMN IC in bilateral PCC/Pcu, while no significant decreased functional connectivity of DMN IC was observed in aMCI subjects at baseline (Table 3, Figure 3-1). In addition, there was evidence of a greater decrement in functional connectivity of DMN IC in the aMCI group compared to controls group in several regions (Table 3, Figure 3-2). Areas identified were bilateral PCC/Pcu and right anterior cingulate/ medial frontal gyrus. It should be noted that PCC/Pcu were associated with the most extensive progressive deficits in aMCI subjects (i.e. cluster size and mean T values, Figure 3-2). Interestingly, there was no significant change of prefrontal cortex in baseline aMCI subjects, while increased functional connectivity of the prefrontal cortex was observed at follow up in aMCI subjects compared with controls, including bilateral superior frontal gyrus/ middle frontal gyrus, left middle/inferior frontal gyrus. In addition, there was no evidence on less longitudinal changes of DMN IC in aMCI compared with controls.

**Table 3:** Post hoc test: abnormal functional connectivity of DMN IC in aMCI compared with controls.

Brain region	BA	Peak MNICoordiates x, y, z (mm)	PeakT value	cluster size
**(1) Increased functional connectivity of DMN IC in aMCI compared with controls at baseline**				
R Posterior Cingulate Cortex/ Precuneus	30/31	3 -57 12	4.95	7641
*L Posterior Cingulate Cortex/ Precuneus*	*30*	*-3 -63 9*	*4.75*	
**Decreased functional connectivity of DMN IC in aMCI compared with controls at baseline**				
None				
**(2) Greater longitudinal increases of DMN IC in aMCI compared with controls**				
L Superior Frontal Gyrus/ Middle Frontal Gyrus	6/8	-9 27 57	6.33	13878
*R Superior Frontal Gyrus/ Middle Frontal Gyrus*	*6/8*	*9 27 54*	*5.49*	
L Middle Frontal Gyrus	10	-42 39 3	4.56	3132
L Inferior Frontal Gyrus	47	-54 36 -3	5.73	4833
**Greater longitudinal decreases of DMN IC in aMCI compared with controls**				
B Posterior Cingulate Cortex/ Precuneus	7/23/29/30/31	9 -66 27	9.44	66366
R Anterior Cingulate/ Medial Frontal Gyrus	10/32	6 48 9	5.00	2997
**Less longitudinal changes of DMN IC in aMCI compared with controls**				
None				

Note: A corrected threshold by Monte Carlo simulation at *P*<0.05. R =  right; L =  left; B =  Bilateral; BA =  Brodmann’s area; secondary peaks are italic; Cluster size is in mm^3^; MNI: Montreal Neurological Institute.

### DMN dysfunction and behavioral significance

The common regions associated with baseline changes and longitudinal changes of DMN IC were in PCC/PCu in aMCI group compared to controls (Figure 3–3). Particularly, hyper-functional connectivity between the PCC/PCu and mean DMN IC in baseline aMCI subjects, and significant hypo-connections of these regions were in follow-up aMCI subjects, whilst controls only showed a light decreases at the longitudinal period (Figure 3–4). Furthermore, the decreases of functional connectivity between PCC/PCu and mean DMN IC were positively related to the impairments of episodic memory (AVLT-delayed recall scores, r = 0.462, *P* = 0.018, two-tailed) from baseline to follow up in the aMCI group (Figure 3-5).

## Discussion

This study utilized ICA to investigate spatial patterns of DMN, and to investigate changes in these patterns in aged subjects presenting with aMCI and normally aging participants, over a mean period of 20 months with the objective of determining clinical markers valuable for predicting those aMCI subjects expected to convert to AD in the near future. PCC/PCu hyper-functional connectivity was observed in baseline aMCI subjects, yet a decrement of these connections was far greater in follow-up aMCI subjects, compared to matched controls. Specifically, PCC/PCu dysfunction was positively related to the impairments of episodic memory from baseline to follow up in the aMCI group. This investigative approach may lead to a better understanding of the progressive changes of DMN in aMCI subjects.

Brain imaging research has recently converged to define the brain’s default network - a novel and only recently appreciated brain system that participates in internal modes of cognition [41]. Our previous cross-sectional studies assessing regional homogeneity [29] and seed-based correlational analysis approach [30] have revealed abnormal DMN in aMCI subjects. However, using the ICA method, the current study also confirmed progressive changes of DMN within the aMCI subjects and healthy controls. This change was more prominent in the aMCI subjects, suggesting wide and abnormally rapid deficits of DMN occur in aMCI. It should be noted that the disruption was mainly in parietal cortex and prefrontal cortex. Specifically, these findings may represent a disruption of frontal-parietal network which has been suggested in previous DMN studies of AD and MCI subjects, of task-induced deactivation [19], [27], low-frequency activity fluctuations in the whole brain [18], [24], correlations of intrinsic activity [20]-[23], [25], [26], [28], [29], [31], [49]. Importantly, previous hypotheses have highlighted that DMN directly supports episodic memory processing [9] and other functions [11]. Episodic memory loss is the predominant symptom in aMCI [2], and was also a key deficit in present aMCI subjects. Moreover, the present study provided further evidence of the decreases of DMN dysfunction being positively related to impairments of episodic memory in aMCI. Taking into account previous findings and these study results, DMN may be particularly vulnerable to neuropathology in aMCI patients. Importantly, we found an intriguing functional link between the DMN and aMCI in this longitudinal study. In addition, there was a slight improvement in the performance of aMCI subjects in several cognitive tasks upon follow-up based on visual comparisons (no significant difference in statistics). One potential mechanistic explanation of this slight improvement may be associated with that the brain’s attempt to continually arouse cognitive potentiality in the early development of aMCI, especially as it relates to these aMCI subjects who are still with steady-state clinical diagnosis in short term, and then it is only when this normal mechanism becomes over extended and the underlying deficits will begin to surface. One could argue that the more effective this mechanism is the longer, it disguises the effects of increasing levels of cognition. Thus, when the mechanism eventually falters, a more dramatic level of functional failure is suddenly uncovered which translates into an accelerated decline afterward. However, the details need further study.

A notable finding in this study was the most extensive progressive deficits (i.e. cluster size and mean T values) of functional connectivity between PCC/Pcu and mean DMN IC in aMCI subjects over the study period. It should be noted that the present findings revealed paradoxical hyper-functional connectivity between the PCC/Pcu and mean DMN IC in baseline aMCI subjects, yet significant hypo-connections of these regions were more severely affected in follow-up aMCI subjects, compared to matched controls. However, our previous cross-sectional studies used other approaches, such as decreased regional activation of PCC/Pcu detected by regional homogeneity [29] and decreased functional connectivity of PCC and temporal cortex assessed by seed-based correlational analysis [30] in aMCI subjects. Although the underlying meaning of different measures could be used to interpret the discrepancy of present and previous findings, all these findings highlighted the potential value of PCC/Pcu in the progression of aMCI subjects. Some regions with early hyper-function could be due to compensation for other impairments, indeed the present findings related to increased functional connectivity at baseline were closely associated with a recent study. Bero et al. (2011) suggested that excess activity in the DMN could be amyloidogenic processing through that neuronal activity increases Aβ production and secretion into interstitial fluid [16]. Namely, regional differences in neuronal activity may underlie the spatial relationship between resting-state physiologic function and amyloid deposition in AD. Therefore, DMN increased activity at baseline may be a pathological process that contributes to generating plaques, which then create a pathological load in the same regions that subsequently causes a rapid decline in connectivity in these regions. This was also consistent with the threshold model of AD [50].

Healthy subjects showed the highest regional homogeneity in these regions during the resting state, in which Kendall's coefficient of concordance was used to measure the similarity of time series of a given voxel to those of its neighbors [51]. PCC/Pcu, considered as two of tonically active regions of the resting brain with high metabolic rates, is identified as an anatomic hub in the DMN [52]. Importantly, the PCC/Pcu regions are brain regions associated with the earliest signs of AD-related pathology, as imaging studies have shown that changes in these regions, typically hypometabolism [53], hypoperfusion [54], amyloid deposition [55], volume reduction [56], reduction in regional homogeneity [24], [29], activation [19], [27], and functional connectivity [20], [25], [26], [28], [30], [31] were associated with both AD and MCI patients. Another marked finding was that the hysteretic increased functional connectivity between the prefrontal cortex and mean DMN IC was observed in aMCI subjects compared with controls at follow up. The present study supported that the abnormal changes of PCC/Pcu maybe presented at an earlier stage than the changes occurring in prefrontal cortex in aMCI subjects. Therefore, it was not surprising that the deficits were predominantly observed in PCC/Pcu in MCI subjects, whilst AD patients almost showed concurrent changes in these regions [19], [20], [24]–[31], [53]–[56], albeit the details still need to be further explored. Therefore, this is crucially important if treatment options and delivery are to be best distributed across the at risk population.

There were technical and biological limitations in the present study which must be acknowledged. Firstly, there is a considerable clinical and biological heterogeneity in samples of present aMCI subjects whose recruitment were based only on clinical criteria. Some subjects may not display the underlying AD-pathology, and represent a ‘contamination’ of the sample with non-AD cases. This could be obtained by means of adding biomarker information (CSF, PET or structural MRI data) to better characterize the study groups, albeit our previous study also observed significant atrophy of hippocampus in aMCI subjects compared to controls (It should be noted that only some subjects of the present samples were recruited in our early-stage study) [57]. This would further yield the current diagnosis of ‘aMCI due to AD’, as recently published in the revised diagnostic criteria for AD [58]. Secondly, no conversions from aMCI to dementia were directly follow-up scanned, the present findings based on two time points of aMCI should be interpreted with caution in the prediction of disease's development, especially it may relate to some aMCI subjects referring to the possibility of ‘reverse conversion’ or ‘long-term steady state’ in the future. Thirdly, the previous studies have demonstrated only moderate test-retest reliability of resting state fMRI measures [59], [60]. Therefore, replication of these findings in larger cohorts will be necessary for validation. Finally, the present aMCI subjects included aMCI-single domain and aMCI-multiple domain, and precise subgroups could be recommended in future study, such as single domain and multiple domains, separately. Despite these limitations, these findings may have important clinical implications, as this investigative approach may lead to a better understanding of the progressive functional neurodegeneration underlying this disease and a possible means to monitor development of aMCI subjects who are with high risk of AD.
